# Formulation, Optimization, *In Vitro* and *In Vivo* Evaluation of Saxagliptin-Loaded Lipospheres for an Improved Pharmacokinetic Behavior

**DOI:** 10.1155/2021/3849093

**Published:** 2021-10-20

**Authors:** Akhtar Rasul, Safirah Maheen, Hafeez Ullah Khan, Maria Rasool, Shahid Shah, Ghulam Abbas, Khurram Afzal, Fatima Tariq, Irum Shahzadi, Muhammad Hassham Hassan Bin Asad

**Affiliations:** ^1^Department of Pharmaceutics, Faculty of Pharmaceutical Sciences, Govt. College University Faisalabad, Faisalabad, Pakistan; ^2^Department of Pharmaceutics, College of Pharmacy, University of Sargodha, Sargodha, Pakistan; ^3^College of Allied Health Professionals, Faculty of Medical and Allied Sciences, Govt. College University Faisalabad, Faisalabad, Pakistan; ^4^Department of Pharmacy Practice, Faculty of Pharmaceutical Sciences, Govt. College University Faisalabad, Faisalabad, Pakistan; ^5^Institute of Food and Nutrition, Bahauddin Zakariya University, Multan, Pakistan; ^6^Department of Biotechnology, COMSATS University Islamabad, Abbottabad Campus, 22060, Pakistan; ^7^Department of Pharmacy, COMSATS University Islamabad, Abbottabad Campus, 22060, Pakistan; ^8^Institute of Fundamental Medicine and Biology, Department of Genetics, Kazan Federal University, Kazan, Russia

## Abstract

The development and optimization of controlled release lipospheres (LS) from safe biocompatible behenic acid (BA) was performed for not only enhancing patient's compliance against highly prevailed chronic diabetes but also to vanquish the insufficiencies of traditional methods of drug delivery. The Box-Bhenken design (BBD) was utilized to statistically investigate the impact of formulation variables on percentage yield (*Y*_1_), entrapment efficiency (*Y*_2_), and SG-release (*Y*_3_) from saxagliptin- (SG-) loaded LS, and the chosen optimized LS were subjected to a comparative *in vivo* pharmacokinetic analysis against commercially available SG brand. The compatibility analysis performed by DSC and FTIR established a complete lack of interaction of formulation components with SG, while p-XRD suggested a mild transformation of crystalline drug to its amorphous form during encapsulation process. The spherical, free flowing smooth surface LS having zeta potential of -32 mV and size range of 11-20 *μ*m were conveniently formulated. The obtained data for *Y*_1_ (30-80%), *Y*_2_ (30-70%), and *Y*_3_ (40-90%) showed a best fit with quadratic model. The pharmacokinetics analysis of LS showed a significantly decreased *C*_max_ of SG (75.63 ± 3.85) with a sufficiently elevated *T*_max_ (10.53 h) as compared to commercial brand of SG (99.66 ± 2.97 ng/mL and 3.55 ± 2.18 h). The achievement of greater bioavailability of SG was most probably attributed to higher level of half-life, mean residence time (MRT), and AUC_0-24_ for SG released from LS. Conclusively, the novel approach of SG-loaded LS had successfully sustained the plasma SG level for a prolonged time without increasing *C*_max_ which would ultimately bring an effective management of chronic diabetes.

## 1. Introduction

Diabetes mellitus (DM) has been proclaimed as the fifth foremost cause of death and is expected to affect 592 million people up to 2035 as reported by International Diabetes Federation. Most common type of DM is type-2 characterized by an increase glucose production, impaired insulin secretion, and variable degree of insulin resistance [1]. Although several medications are available for DM, successful cure is still not achieved because of occurrence of several adverse effects like transient nausea, gastric irritation, and injection phobia caused by these treatments which eventually lead to patients' noncompliance [2]. The pharmacological therapy is usually recommended after failure of lifestyle modification for those diabetics who are unable to maintain their blood glucose level [1]. The conventional therapies to treat existent diabetes consist of complex drug regimen that leads to increased pill burden, frequent and high daily dosing, and dose-associated side effects and ultimately results in poor patients' compliance with worsening of such patients' condition. So, the idea of sustained delivery of an antidiabetic agent was generated because the mode of drug administration has been contributing a lot in improving therapeutic efficacy and decreasing adverse effects of a drug. So, to overcome noncompliance, discovery of novel controlled release methods of drug delivery like multitablet system, slowly eroding hydrophilic matrix system, microparticulate, lipospheres, coated beads, and complex formation for delivery of drug has been proven to be more beneficial [3].

Microparticulate system works by controlling the release rate of drug, so that therapeutic concentration of drug with shorter half-life is maintained in plasma for longer duration [4]. There are several categories of microparticulate systems such as metal-based, biological microparticles, polymeric microparticles, and lipospheres [4, 5]. Lipospheres also known as solid lipid microparticles are the formulations that can administer biological, synthetic, and semisynthetic drugs [6]. Lipid-based polymers like fatty acids and waxes have been used for formulation of lipospheres as narrated in literature for antidiabetic drugs by Bhosale et al. for the encapsulation of pioglitazone in compritol 888 ATO and Shivakumar et al. for encapsulating glipizide in stearic acid and paraffin wax [7, 8]. Lipospheres can enhance patient's compliance by reducing drug dosing frequency, lowering adverse effects, and limiting fluctuation in plasma drug levels. Size of lipospheres varies from 1 to 1000 *μ*m, and various methods like solvent evaporation, spray drying, ionotropic gelation, coacervation, and melt emulsification congealing method have been employed for lipospheres preparation [9]. Lipids have exhibited efficient biodegradability, biocompatibility, and ease of utilization in production process due to their lower melting points [10]. Liposphere production and their characteristics are greatly affected by different factors like microencapsulation technique, type and concentration of lipid, concentration and type of surfactant, stirring time, and stirring speed [11, 12].

Saxagliptin (SG) is a potent antidiabetic specifically designed for the extended inhibition of DPP-4 (dipeptidyl peptidase-4) enzyme [13]. The SG is available in 2.5 mg dose having poor membrane permeability which ultimately limits the oral bioavailability of SG up to 50%. The solubility of SG in water may further lead to the short elimination half-life (4-6 h), thus demanding more than once a daily dosing. Moreover, the uncontrolled release from conventional tablets and higher systemic concentration of SG might also be responsible for gastrointestinal disturbances like nausea, vomiting, diarrhea, stomach pain, and bloating. Saxagliptin is also a substrate for the P-glycoprotein driven efflux that also contributes in rapid removal of drug. Thus, controlled release LS may prove to be a good choice to solve the aforementioned drug problem and may enhance patient's compliance. The SG belongs to BCS class III, and several therapeutic agents like metformin, chloromphenical, didanosine, and ethambutol belonging to BCS class III had been formulated into LS for administration through oral, ophthalmic, buccal, and pulmonary routes [3, 8, 11]. For the first time, current study is aimed at formulating a sustained release lipid-based system for administration of SG for chronic illness, and the study may got the potential to enhance treatment effectiveness for type-2 diabetic patients.

The behenic acid (BA) was utilized as the drug release retardant for preparing controlled release SG-loaded lipospheres (LS) by hot melt emulsion congealing technique. The insoluble nature of lipids makes them ideal carriers for developing the controlled release particulate systems like LS, and the BA also presents the property of being biodegradable with a melting point of 80°C making it ideal candidate for encapsulating SG. The study has a focus on the comparison of selected SG-loaded OL with the commercially available brand of saxagliptin based on their *in vivo* pharmacokinetic behaviour in healthy male volunteers.

## 2. Materials and Methods

### 2.1. Materials

Saxagliptin was generously gifted by Getz Pharma (Pvt) Ltd. Karachi, Pakistan. Tween 80 and potassium dihydrogen phosphate were purchased from Merck, Germany. Behenic acid (Sigma Aldrich, USA) and potassium bromide (Fischer Scientific, UK) of IR grade cellulose dialysis tube (Sigma-Aldrich, USA) were also purchased. All the used chemicals and reagents were of analytical grade.

### 2.2. Preparation of Lipospheres by Hot Emulsion Congealing Technique

Liposphere formulations were prepared by the hot emulsion congealing technique. An aqueous solution of Tween-80 (T-80) was prepared and maintained at 75°C. A specific amount of drug was dissolved in melted BA at 80°C which was then added to hot aqueous surfactant solution along with continuous homogenization at 1500, 2250, and 3000 RPM for 30 min by employing a high-speed homogenizer (Yellow Line Ost Basic Company, Germany). Afterwards, the obtained preemulsion was then directly conveyed to an ice-box to initiate generation of LS. The yielded LS were allowed to recrystallize at room temperature, and the obtained formulation was then separated by centrifugation at 5000 rpm for 40 minutes at 3-4°C. The separated LS were then subjected to lyophilization (Marya Pharmaceutical Turnkey, Model 200121, China) process for 24 h to produce completely dried LS. This approach was repeated by changing three factors to obtain all formulations (Table 1) including the selected optimized one by keeping the ratio of aqueous phase volume (500 mL) and drug concentration (50 mg) constant [14, 15].

A response surface methodology known as Box-Bhenken design was applied for designing and optimizing SG-loaded LS formulations [13–15] by a statistical software Design Expert (version 8.0.6.1 Stat-ease, Inc. USA). The determination of compatibility of drugs with the polymer was carried out by DSC, FTIR, and XRD studies. The evaluation for particle size, micromeritics behavior, morphological appearance, and zeta potential was also performed. To assess the *in vitro* release profiles of SG, various mathematical kinetic models like Higuchi, zero-order, first-order, and Korsmeyer Peppas models were utilized. The extensive statistical investigation of influence of formulation factors—BA concentration (*X*_1_), T-80 amount (*X*_2_), and stirring speed (*X*_3_)—on three decided responses like percentage yield (*Y*_1_), encapsulation efficiency (*Y*_2_), and SG release (*Y*_3_) from LS was also a part of current study. For creating optimum conditions to attain optimum intensity of each studied response, numerical optimization technique was applied which helped in selection of optimized lipospheres formulation (OLF).

### 2.3. Technique to Select Optimized LS Formulation (OLF)

After a complete analysis of the suggested seventeen formulations by BBD, then numerical optimization technique was employed to establish suitable conditions for formulation variables (*X*_1_, *X*_2_, *X*_3_) in order to achieve desirable outcomes of studied responses (*Y*_1_, *Y*_2_ and *Y*_3_). The studied responses were thoroughly analyzed on the desirability scale (0-1) by the software design expert (version 8.0.6.1 Stat-ease Inc., USA). By running a detailed feasibility investigation, then selection of optimized conditions was made for synthesizing optimized liposphere formulation (OL) in order to achieve maximum percentage yield (90%), maximum EE (80%) but minimum SG release (<40%) because the control of drug release usually remained a critical factor for designing a controlled release drug delivery system. The recommended OL was finally prepared and characterized not only for the *in vitro* analysis but also for the detailed pharmacokinetic analysis in healthy volunteers. A comparative analysis of observed and predicted outcomes of three studied responses for OLF was also made to find prediction error (PE) by the following equation:

(1) $$\begin{array}{c} \text{PE }\left(\%\right)=\left[\left(\text{Experimental value}-\text{Predicted value}\right)/\text{Predicted value}\right]\times 100. \end{array}$$

### 2.4. HPLC Method Development

For the quantification of SG present in LS and in blood, an HPLC method was devised and validated in mobile phase and in plasma for the purpose of selection of suitable mobile phase along with optimization of flow rate. The designed method was further validated as per ICH guidelines in both the mobile phase and plasma where correlation coefficients (*R*^2^) for SG were also found in linearity analysis, and calculation of interday and intraday precisions was also performed. The % recovery of drugs and determination of LOD and LOQ were performed with calculation of %RSD for all the HPLC process validating parameters [16]. In this study, the high-performance liquid chromatographic system consisting of HPLC pumps (LC-10AT and LC-10AT VP Shimadzu, Japan,) having a manually operated 20 *μ*L sample loop and UV-visible detector (SPD 10A VP) was used. The output signal was integrated by Shimadzu CLASS-VP version 6.12 SP1 software. Before running mobile phase through column-C18 (250 mm × 4.6 mm, 5 *μ*m particle size (Sigma-Aldrich, Darmstadt, Germany)), the mobile phase was first filtered through 0.22 *μ*m membrane filter, and mobile phase was consistently run through system for 20 minutes prior to inject the sample solution to equilibrate the system.

### 2.5. Evaluation of SG-LS

#### 2.5.1. Micromeritics Studies of LS

In micromeritics studies, the flow behavior of LS formulations was analyzed. The appropriate flow behavior of LS is considered as an essential parameter particularly when LS have to be transformed into capsule, tablet, or any other dosage form. The micromeritics parameters like Hausner's ratio and Carr's index were calculated from bulk and tapped densities, and the angle of repose was also calculated by using funnel method. Equations (2)–(4) for calculation of micromeritics parameters were given below. The value of Carr's index ranging from 13% to 21% is an expressive of good flow character, while a Hausner′s ratio < 1.25 is usually desirable, and the angle of repose < 30° indicates free flow behaviour of LS [17].

Carr's index

(2) $$\begin{array}{c} I=\frac{V_{b}-V_{t}}{V_{t}}\times 100, \end{array}$$

For Hausner's ratio

(3) $$\begin{array}{c} \text{H}.\text{R}=\frac{\rho t}{\rho b}, \end{array}$$

For Angle of repose

(4) $$\begin{array}{c} \text{Tan}\theta =\frac{h}{r}. \end{array}$$

#### 2.5.2. Compatibility Analysis

The compatibility of SG with BA was analyzed by FTIR (IR Prestage 21 Shimadzu Japan) spectroscopy, and the FTIR spectra of drug alone, lipid (BA) alone, and SG-loaded optimized lipospheres (OL) were recorded at a resolution of 2 cm^−1^ by maintaining scanning range at 400-4000 cm^−1^ with hydraulic pressure of 150 kg/cm^2^. Discs for analysis were prepared by mixing IR grade KBr with sample (1 mg sample and 100 mg KBr). The compatibility analysis was further extended by applying differential scanning calorimetry (DSC). Thermal analyzer (SDT Q600 TA USA) was used for analyzing the drug-lipid interaction. For DSC analysis, SG, BA, and SG-loaded lipospheres were finely triturated, and the sample was then subjected to heating at a rate of 10°C/min from 0°C to 220° C in a sealed aluminum pan. The nitrogen flow was kept at 40 mL/min. Every sample was run in triplicate to check the reproducibility of results [17–19].

#### 2.5.3. Percentage Yield (PY)

For PY, weight of finally prepared dried lipospheres was taken, and obtained weight was divided by total amount of all of the solid components employed to prepare lipospheres. The percentage yield was calculated by following formula [20]:

(5) $$\begin{array}{c} \text{Percentage Yield}=\frac{\text{Weight of Obtained Lipospheres}}{\text{Total Weight of all solid components}}. \end{array}$$

#### 2.5.4. Entrapment Efficiency (EE) of LS

To calculate the amount of SG entrapped in lipospheres, a specific amount of lipospheres was crushed and dispersed in a phosphate buffer (pH 6.8) along with continuous stirring for 24 hrs. Sample was taken, filtered, and diluted by mobile phase and then injected in HPLC after filtration in order to calculate encapsulated amounts of SG in LS by following formula [16, 20]:

(6) $$\begin{array}{c} \text{EE}=\frac{\text{Actual drug amount}}{\text{Theoratical drug amount}}\times 100. \end{array}$$

#### 2.5.5. In Vitro Drug Release Study

The drug release from the liposphere formulations was evaluated for duration of 12 hrs in phosphate buffer (pH 6.8) as a dissolution medium (900 mL) at 37 ± 0.5°C with a rotating speed of 50 rpm by employing USP type-II dissolution apparatus (PT-DT7, Pharma Test Germany). For each formulation, sample of lipospheres equivalent to 5 mg of SG was added in a cellulose dialysis tube having 5 mL of dissolution medium. This tube was attached with paddle in order to maintain sink condition in dissolution vessel. After a specific interval of time (30 minutes), a 5 mL sample from each dissolution vessel was withdrawn, and an equal volume (5 mL) of freshly prepared prewarmed dissolution medium was added in the same vessel. The unknown concentration of SG in samples was determined by already established and validated HPLC method after diluting the obtained sample with mobile phase [15, 16, 19]. The *in vitro* drug release study was performed in triplicate for every formulation.

#### 2.5.6. Drug Release Kinetics

To study the drug release mechanism, analysis of release data was carried out with the help of different drug release kinetics models like first-order, zero-order, Higuchi, Korsmeyer-Peppas, and Hixson-Crowell models which were as follows [19]:

(7) $$\begin{array}{c} F_{t}=K_{0}t, \end{array}$$

(8) $$\begin{array}{c} \text{Log}F=\log F_{0}-\frac{K_{t}}{2.303}, \end{array}$$

(9) $$\begin{array}{c} F=K_{H}t^{1/2}, \end{array}$$

(10) $$\begin{array}{c} F_{0}^{1/3}-F_{t}^{1/3}=K_{HC}\times t, \end{array}$$

(11) $$\begin{array}{c} \frac{M_{t}}{M_{\infty }}=K_{3}t^{n}. \end{array}$$

First-order and zero-order rate constants were represented by *K*_1_ and *K*_0__,_ respectively. Hixson-Crowell rate constant was denoted by *K*_HC_ and *K*_H_ denoted Higuchi rate constant. Initial drug concentration was denoted by *F*_0_ and the concentration of drug at time *t* was indicated by *F*_*t*_. Korsmeyer-Peppas rate constant was represented by *K*_3_, while exponent of drug release mechanism was denoted by *n*. Fickian mechanism was considered if value of *n* < 0.43, whereas greater than 0.43 and less than 0.85 value of *n* denoted non-Fickian drug release mechanism. However, the values of *n* greater than 0.85 propose slow erosion plus diffusion drug release mechanism [19].

#### 2.5.7. Zeta Potential and Particle Size Measurements

The charge on liposphere formulation was examined by measurement of electrophoretic mobility of lipospheres in U-shaped tube at 25°C with the help of Malvern Zetasizer (Zetasizer Ver System; Malvern Instruments Ltd., Malvern, UK). Distribution of size and average size of drug-loaded optimized lipospheres (OL) were also measured. For size measurement and polydispersity index (PDI), 10 mg of LS were suspended in 10 mL of deionized ultrapure water containing 0.01% T-80. The glass cuvette containing suspension was placed in dynamic light scattering Zetasizer for necessary measurements [21].

#### 2.5.8. Surface Morphology of LS

Scanning electron microscope (JSM-840, Joel Instruments, Tokyo, Japan) was employed to examine the shape and surface morphology of the optimized lipospheres (OL). Liposphere sample was placed on a double-adhesive tape in drops form which was then struck to an aluminum stub. Prepared sample was evaporated, and after that, aluminium stubs were subjected to coating with gold to make them conductive under an argon atmosphere. The photomicrographs of lipospheres were then taken and observed [15].

#### 2.5.9. X-Ray Powder Diffraction Studies

In order to examine the impact of melt emulsion congealing microencapsulation process on drug crystallinity, XRD studies were performed. With employing D8 advance X-ray diffractometer (Bruker AXS, Madison, WI, USA), samples were subjected to irradiation monochromatized X-rays of Cu-K*α* at a current of 40 mA by using rays with a voltage of 40 kV. Scanning was conducted at a scan rate of 2° min^−1^ in the diffraction angel (2*θ*) range from 0° to 65° of the samples like SG, BA, and SG-loaded optimized lipospheres [8, 21, 22].

#### 2.5.10. Stability Studies

The OLF was subjected to a stability study for 3 months to know the influence of environmental condition on drug contents, size, and PDI of LS. The optimized LS were stored in the airtight amber glass bottles at temperature of 25 ± 3°C and at refrigeration temperature of 4 ± 2°C with a relative humidity of 65 ± 5% in stability chamber for 3 months. To evaluate OLF stability, SG amount, LS size, and PDI were examined thrice after a period of every thirty days.

#### 2.5.11. Pharmacokinetic Evaluation of Optimized LS

For pharmacokinetic evaluation of OLF and marketed brand of SG in healthy male volunteers, the protocol was planned in total compliance with declaration of Helsinki and was approved by the Research Institutional Review Board of Faculty of Pharmacy, Government College University Faisalabad, Punjab, Pakistan (IRB no-4145 approved at April, 2020, for a period of eight months). Before study, an informed written consent was obtained from nonsmokers 25-35 years old male volunteers having good physical, mental health and with normal kidney and liver functions. Twelve healthy male participants were randomly divided into two groups having six members in each group. All participants (volunteers) of study were made fully informed about the nature, aim, and scope of the study. All the volunteers were advised to refrain themselves from any medicines and smoking for at least five days before initiation of study and must observe the instructions till the completion of study. At night, volunteers fasted for at least 10 h and after 2 h of administration of treatment at morning, breakfast was provided to volunteers.

It was a noncompartmental, single-dose, open-labelled, parallel pharmacokinetic study design, where the reference employed was the marketed brand of SG to compare pharmacokinetic parameters after administration of single equal oral dose to participants of group. The parallel pharmacokinetic study design was adopted because the crossover design usually designed and based on the outcomes of parallel study design. Moreover, the parallel design is considered as most common and basic design where the volunteers remained adhered to study protocol till its completion because of ease and short duration of this study design. A specified amount of OLF containing 5 mg of SG was filled in hard gelatin capsule shell, and the capsule was swallowed by the healthy volunteers with 250 mL water. The blood samples (1.5 mL) at prespecified time points of 0, 2, 4, 6, 8, 10, 12, 16, 20, and 24 h were obtained into heparinized tubes, and volunteers were supervised entirely for safety concerns during sample collection [23]. For extracting SG from plasma, acetonitrile was added to plasma sample (200 *μ*L), vortexed for about 20 min, and then centrifuged at 15000g for 30 min. The obtained supernatant layer of organic solvent was separated and subjected to a nitrogen stream for 25 minutes at 40°C to remove acetonitrile. Resulting powder extract was reconstituted with mobile phase, and plasma SG level was analyzed by HPLC method. The pharmacokinetic variables like AUC (ng/mL.h), *T*_max_ (h), *C*_max_ (ng/mL), MRT (h), *k*_el_ (h-1), and *t*_1/2_ (h-1) for SG brand and OLF were calculated by using PK solver, a freely available menu driven add-in program for Microsoft Excel 2016 [24] and statistically analyzed by using one-way ANOVA.

### 2.6. Statistical Analysis

The polynomial equations and suggested statistical model for every response were validated statistically with the help of ANOVA. Moreover, each response and data model were further analysed with respect of predicted residual sum of square (PRESS), coefficient of variation (CV), the correlation coefficient (*R*^2^), and the adjusted correlation coefficient (adjusted *R*^2^). For assessing the antagonistic or synergistic effects of independent variables on studied responses, 3D surface plots and comparative plots of predicted and actual values were also plotted [20, 25, 26].

## 3. Results and Discussion

Different formulations and their components, percentage yield, entrapment efficiency, and drug release profile of lipospheres are summarized in Table 1. A mathematical relationship in the form of polynomial equations between responses (*Y*_1_, *Y*_2_, *Y*_3_) and independent factors (*X*_1_, *X*_2_, *X*_3_) was generated to indicate the effects of formulation variables alone and their interaction on responses. The generated polynomial equations by software design expert for *Y*_1_: PY, *Y*_2_: EE, and *Y*_3_: drug release at 12 h were depicted below;

(12) $$\begin{array}{c} \text{Percentage Yield}=+60.03-35.33X_{1}+17.65X_{2}+3.73X_{3}+8.08X_{1}X_{2}+4.76X_{1}X_{3}+11.22X_{2}X_{3}+2.53X_{1}^{2}-17.02X_{2}^{2}-3.48X_{3}^{2}, \end{array}$$

(13) $$\begin{array}{c} \text{Entrapment Efficiency}=+59.66+38.75X_{1}+18.75X_{2}+1.78X_{3}-11.54X_{1}X_{2}-3.24X_{1}X_{3}+5.45X_{2}X_{3}-6.06X_{1}^{2}+3.04X_{2}^{2}-2.53X_{3}^{2}, \end{array}$$

(14) $$\begin{array}{c} \text{SG Release}=+114.03+14.72{\text{X}}_{1}-61.95{\text{X}}_{2}-8.32{\text{X}}_{3}+12.00{\text{X}}_{1}{\text{X}}_{2}+7.73{\text{X}}_{1}{\text{X}}_{3}-2.35{\text{X}}_{2}{\text{X}}_{3}-3.15{\text{X}}_{1}^{2}-32.42{\text{X}}_{2}^{2}+2.62{\text{X}}_{3}^{2}. \end{array}$$

**Figure 1:**
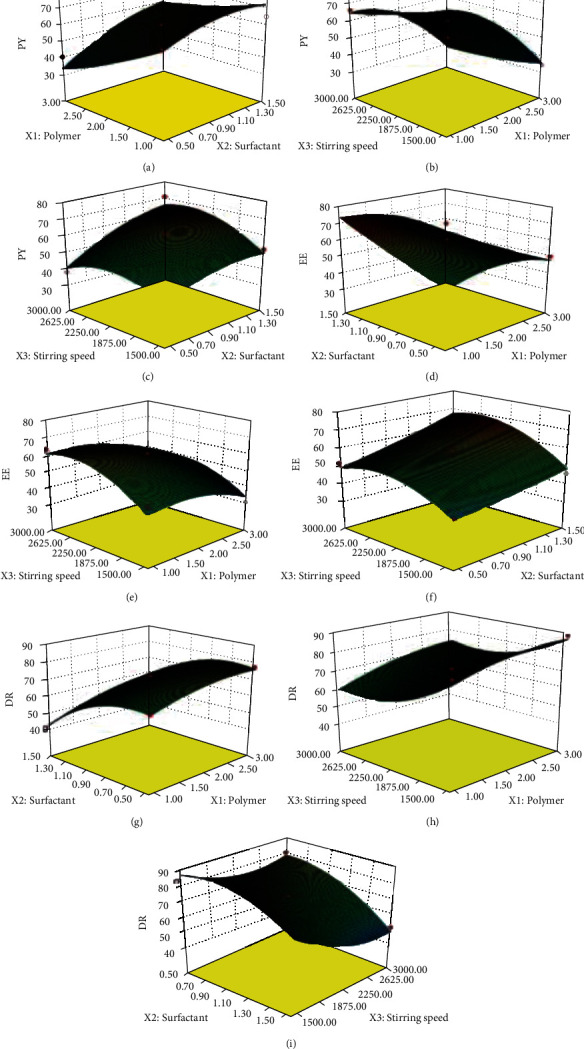
3D response surface plots indicating the collective effect of behenic acid (*X*_1_) plus T-80 (*X*_2_) on percent yield (a), behenic acid (*X*_1_) plus stirring speed (*X*_3_) on percent yield (b), T-80 (*X*_2_) plus stirring speed (*X*_3_) on percent yield (c), behenic acid (*X*_1_) plus T-80 (*X*_2_) on entrapment efficiency (d), behenic acid (*X*_1_) plus stirring speed (*X*_3_) on entrapment efficiency (e), T-80 (*X*_2_) plus stirring speed (*X*_3_) on entrapment efficiency (f), behenic acid (*X*_1_) plus T-80 (*X*_2_) on drug release at 12 h (g), behenic acid (*X*_1_) plus stirring speed (*X*_3_) on drug release at 12 h (h), and T-80 (*X*_2_) plus stirring speed (*X*_3_) on drug release at 12 h (i).

In order to statistically validate the model and polynomial equations, analysis of variance (ANOVA) was applied at 5% significance level with design expert software, and three-dimensional surface graphs depicting the effect of factors (*X*_1_-*X*_3_) on responses were presented in Figure 1.

Results of responses (*Y*_1_, *Y*_2_, and *Y*_3_) ranged from 32 to 70%, 30 to 70%, and 42 to 90%, respectively, as presented in Table 1. The ratio of maximum to minimum for *Y*_1_, *Y*_2_, and *Y*_3_ was found to be 2.1, 2.33, and 2.14, respectively, indicating that there was no need for further alteration of model as it was found to be <3, and the power transformation would have little effect on model validation. A *p* value < 0.05 represented that model was significant. Quadratic model was followed by all three responses because they indicated a good fit relation with quadratic model instead of linear model. The quadratic model was also evaluated for LOF (lack of fit) with the help of Design Expert software, and it was not found to be significant.

The predicted *R*^2^ values for *Y*_1_ (0.8568), *Y*_2_ (0.7653), and *Y*_3_ (0.7929) were observed to be very close to the values of adjusted *R*^2^ of *Y*_1_ (0.7893), *Y*_2_ (0.8425), and *Y*_3_ (0.9554), respectively, suggesting the suitability of quadratic model for studied responses. Measurement of signal to noise ratio was done from adequate precision, and its values for *Y*_1_ (21.638), *Y*_2_ (25.314), and *Y*_3_ (23.067) were observed to be greater than 4 (Table 2) indicating the suitability and adequacy of model for all three responses [22].

Percentage yield of lipospheres was calculated for all liposphere formulations, and results indicated that PY had been significantly influenced by BA and surfactant (T-80) concentrations and stirring speed. A significant variation of *Y*_1_ was observed from 32% (F3) to 70% (F1). Use of higher amount of BA with lower concentration of surfactant like in formulations F2, F3, F8, F14, and F15 showed a PY of <50%. The lower yield of lipospheres could be due to aggregation of lipid polymer because of less stabilization of lipid droplets at lower concentrations of T-80 [19]. However, using higher concentration of T-80 along with higher amount of BA like in formulations F1, F6, and F17 was found to be a good condition because results indicated greater than 65% percentage yield (Table 1). Similar results were also published in literature for biodegradable microparticulate system [20].

The equation attained for *Y*_1_ (Equation (12)) showed that *X*_1_ had a negative effect on PY which indicated that increase in BA concentration alone leads to a decline in PY. Quadratic model was shown to be significant for *Y*_1_ as the *F* value for *Y*_1_ was 11.43 (*p* < 0.0001) showing significance of model. For *Y*_1_, the *p* value of *X*_2_ was <0.0001 indicating that *X*_2_ (T-80 concentration) was also a significant term. Concentration of surfactant played a dynamic role in the emulsion droplets stability and development which finally contributed towards an increased PY of lipospheres. The yield was observed to be very low at lower T-80 concentrations because higher BA level along with lower concentration of surfactant leads to accumulation and aggregation of polymer instead of production of lipospheres. The two-way interactions between BA and surfactant (*X*_1_*X*_2_) and between surfactant and stirring speed (*X*_2_*X*_3_) were found to be not only positive but also significant for PY as indicated in Table 3 and Equation (12). For *Y*_1_, the *p* values for *X*_2_ and *X*_3_ were 0.0014 and 0.0019 indicating that both terms were significant as shown in (Table 3), and other significant terms were *X*_3_, *X*_2_^2^, and *X*_3_^2^, while for *Y*_2_, *X*_1_, *X*_2_, *X*_3_, *X*_1_^2^, *X*_2_^2^, and *X*_3_^2^ were found to be significant terms as indicated in Table 3. Similarly, the *p* values for *X*_1_-*X*_3_ were less than 0.0001 indicating that these three factors have significant effect on *Y*_2_ (entrapment efficiency) [23].

Quadratic model was also followed by *Y*_2_ (EE), *Y*_3_ (DR), and regression equations (Equations (13) and (14)) were obtained. Polynomial equations were statistically validated for *Y*_2_ and *Y*_3_ with the help of ANOVA with a level of significance (*p*) <0.05. The *F* value for *Y*_2_ and *Y*_3_ was found to be 9.36 and 39.08, respectively, which further proved the model significance. Entrapment efficiency (*Y*_2_) for each lipospheres formulation was analyzed, and it was found to be varied from 30% (F15) to 70% (F12) as shown in Table 1. An increase in BA concentration along with increase in surfactant T-80 concentrations brought an equal increase in EE as observed for F5, F9, F11, F13, and F16 but after a certain limit, further increase in BA concentration showed a decline in EE as observed for F14 (38%). However, increase in T-80 level with lower concentrations of BA also showed higher EE as observed for F1, F12, and F17 formulations. A positive effect of stirring speed (*X*_3_) was also quite evident on EE as formulations like F1 and F17 had shown EE > 60%. The difference in stirring speed used to formulate LS had led to the difference in EE values of formulations containing same concentrations of polymer and surfactant as observed for F1 processed at 3000 rpm and F10 processed at 1500 rpm. Smaller value of EE was shown by the formulations having lower value of T-80 and processed at lower homogenization speed. An EE of less than 50% had been demonstrated by formulations F3, F6, F10, and F16 having varying concentrations of BA and T-80 but prepared at low stirring speed of 1500 rpm. Formulations formulated with less percentage of T-80 and lower stirring speed like F15 indicated minimum EE (30%). This could happen because of the inadequacy of stirring of lipid droplets which might lead to the coalescence of lipid droplets instead of LS formulation. Formulations F1, F12, and F17 had showed more than 50% entrapment efficiency at a stirring speed of 3000 rpm because this level of speed had improved drug polymer contact time with prevention of sticking of lipid polymer molecules. It was observed that surfactant higher concentrations alone could not increase EE and increase in EE depended on both the enhancement of T-80 concentrations and SS.

Increase in EE could not be attained only by the use of higher concentration of BA because the optimum quantity of T-80 along with stirring speed was also found to be equally important to increase SG entrapment. In the absence of surfactant, the use of BA alone brought aggregation and accumulation of BA in the form of lumps instead of encapsulation of SG in LS formulation. Thus, instead of polymer amount alone, increasing the concentration of surfactant and stirring speed was required to enhance encapsulation efficiency as could be observed in formulations F1 and F12. It was suggested that higher concentration of T-80 could not only bring stability of lipid microparticles in external phase but also prevent drug loss in external phase leading towards higher entrapment of SG in BA.

**Figure 2:**
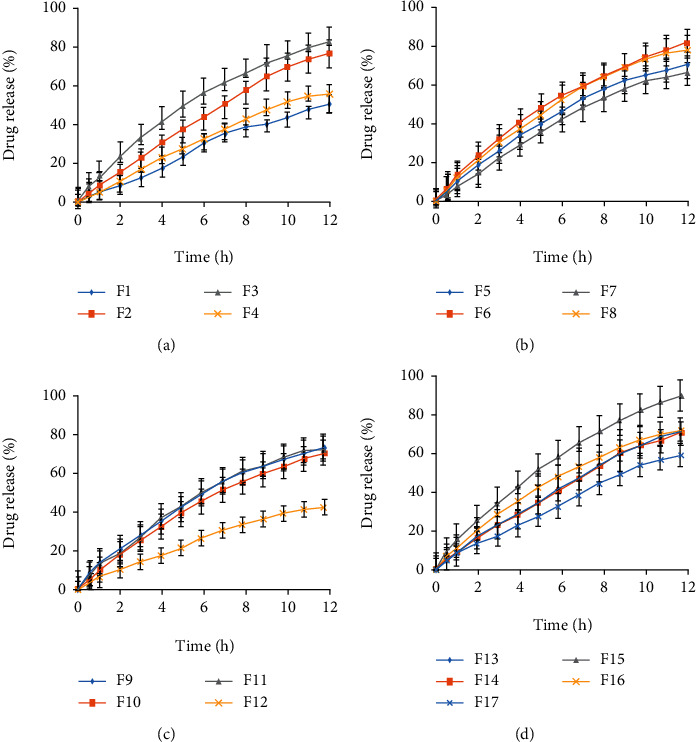
*In vitro* saxagliptin release (%) with respect of time for F1-F4 (a), F5-F8 (b), F9-F12 (c), and F13-F17 (d).

For EE, the two-way interaction of surfactant and stirring speed (*X*_2_*X*_3_) was observed to be significant and positive (Equation (13)) indicating that effective EE could not be achieved in absence of surfactant and stirring speed and alone polymer (BA) had no role in entrapment of SG. All studied two-way interactions like interaction of polymer with surfactant (*X*_1_*X*_2_), interaction of polymer with stirring speed (*X*_1_*X*_3_) and interaction of surfactant with stirring speed were found significant (*X*_2_*X*_3_) for drug release (Table 3). Significant terms for *Y*_2_ were found to be *X*_1_, *X*_2_, *X*_3_, *X*_1_^2^, *X*_2_^2^, and *X*_3_^2^ while on the other hand, *X*_1_, *X*_2_, *X*_3_, and *X*_3_^2^ were found to be significant terms for *Y*_3_ representing their potential effect on EE and drug release (Table 3). For *Y*_1_ and *Y*_2_, *X*_2_ (T-80 concentrations) has positive sign representing positive impact on both responses while for *Y*_3_, *X*_2_ has a negative effect indicating a good control over drug release at 12 h study. Encapsulation of drug was carried out in lipid polymer (BA) to achieve a sustained release action. The release profiles of SG varied from 40% to 90% at pH 6.8 in different liposphere formulations as represented in release graphs (Figure 2).

The graphs represented that drug release from F1 was almost 50% after 12 h because it has 2% polymer-BA and 1.50% T-80, while F13 having 2% of BA and 1% T-80 indicated 90% drug release after 12 h at pH 6.8. Both formulations have same concentration level of polymer but different level of surfactant as F1 has higher concentration of surfactant which ultimately contributed towards controlling drug release by affecting the formulation of lipospheres at production level. Increased proportion of polymer in lipospheres leads to decrease spread and penetration of water molecules in BA which ultimately caused slower drug release. Moreover, increased BA concentrations induce more hydrophobicity to lipospheres. Liposphere formulations (F3, F9, F11, and F13) having lower T-80 concentration like 1.0% offered rapid drug release even more than 70% after 12 h. On the other hand, formulations (F1, F4, and F12) of lipospheres having higher surfactant concentrations (1.50%) presented very slow and sustained release of saxagliptin even less than 50% at pH 6.8 after 12 h.

It was proposed that liposphere preparations controlled the release of SG at intestinal pH. The graphs for drug release (*Y*_3_) indicated a strong association of time of saxagliptin release with lipid and surfactant concentration, and time required for release of drug (*p* < 0.05) was increased with higher concentration of polymer [14, 24] and surfactant. For *Y*_3_, lower levels of lipid, surfactant, and stirring speed could not control or lower the drug release as all three formulation variables were found to be positive in Equation (14). On the other hand, surfactant and stirring speed represented a positive impact on *Y*_3_ at their higher concentrations. Regarding SG release, the LS formulated with decreased level of T-80 showed a faster drug release rate as compared to LS prepared with higher level of T-80. This was thought to be due to formation of cracks and holes on LS surface at lower surfactant concentration which trigger faster SG release. Moreover, instead of LS formulation, a prominent accumulation of BA was quite evident due to less stability of lipid beads at lower concentration of T-80. Formulations F2 and F4 formulated from similar level of BA and stirring speed but exhibited drug release of 77% and 56%, respectively. This fact could only be associated with concentration of T-80 used to formulate F2 (0.5%) and F4 (1.5%). So, it was established that in order to effectively control the drug release, well-formulated LS without holes and cracks is a mandatory condition which would only be possible with sufficient amount of surfactant at interface during microencapsulation process. A greater amount of T-80 would ensure formulation of uniformly smooth surface spherical LS which will in turn ensure highly controlled release of encapsulated drug. While on the other hand, lower level of T-80 would be failed to formulate uniform smooth surface LS which ultimately lead to a fast release of encapsulated drug due to presence of hole and cracks on surface of LS. So, concentration of T-80 was found to be a major contributing factor in controlling the release of drug. Similarly, the higher release rate of drug from F15 could be explained by use of minimum T-80 concentration which might cause cracks and holes on surface of lipospheres. On the other hand, formulation F12 having higher T-80 concentration indicated slower release of drug. So, it was concluded that increased concentrations of T-80 at higher SS might positively retard the drug release at 12 h study (Table 1).

**Figure 3:**
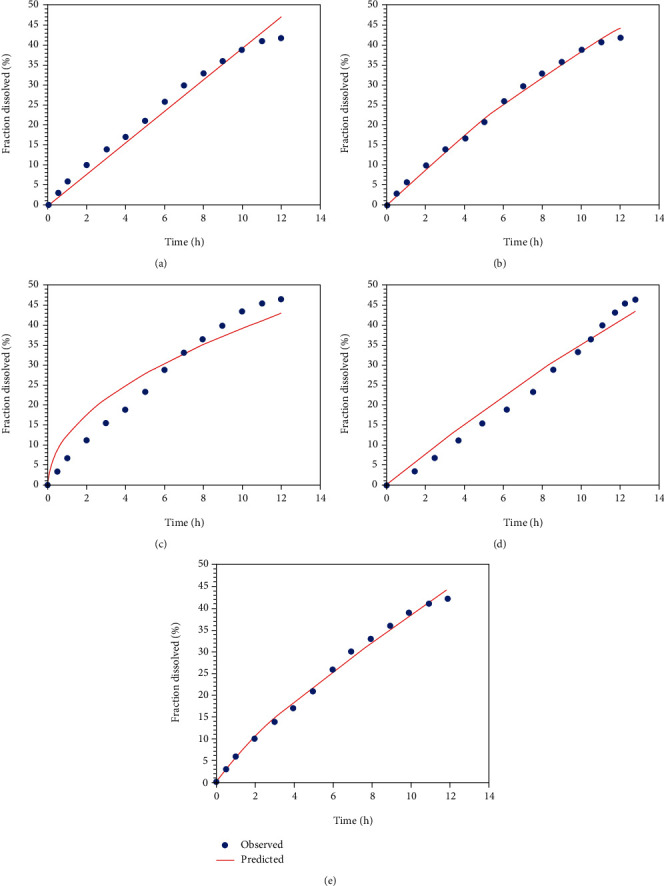
Kinetic modeling of drug release data for zero-order model (a) Higuchi model (b), Korsmeyer peppas model (c), first-order model (d), and Hixon-Crowell model (e).

By using different kinetic models, release profiles of saxagliptin were evaluated. It was evident that data could be better fitted in zero-order model as compared to first-order model, because the values of *R*^2^ of zero order were found to be greater as compared to values obtained from first-order, Haguchi, and Hixson-Crowell models. Sustained release mechanism of saxagliptin release was proposed by obtained data, which represented that release of drug was independent of remaining concentration of drug (Figure 3). Higuchi model was found to be the second best model indicating the diffusion mechanism of drug release [18]. The value of “*n*” was found to be greater than 0.85 as represented by Korsmeyer-Peppas model data, which indicated diffusion along with erosion of lipid polymer [15].

**Figure 4:**
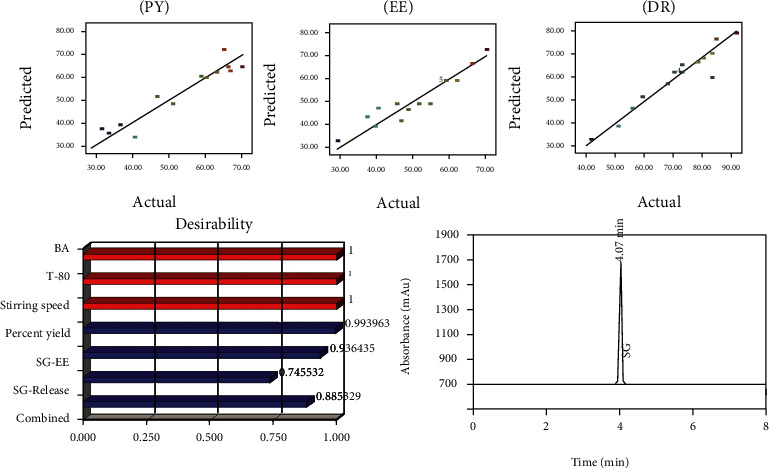
HPLC chromatograph of SG depicting its retention time, predicted versus actual pots, and desirability plot indicating desirability level of PY, EE, and SG release.

According to desirability factor, the suggested solutions for the formulation conditions and the desirable results were prioritized, and the obtained outcomes were presented in desirability plots (Figure 4). The proposed optimized formulation of lipospheres was selected, synthesized, and then further characterized and evaluated. The recommended composition of optimized lipospheres and the observed and predicted results for PY, EE, and SG release are presented in Table 4 along with the calculated prediction errors.

For optimized LS, the desirability factor for all responses was observed to be close to one (Figure 4), and the outcomes of PE were also found to be less than 5% suggesting the validity and success of chosen optimization process [23–25]. Micromeritics studies explain the flow properties of lipospheres, and the results of studies were depicted in Table 5. Although no significant difference could be observed among lipospheres but polymer-surfactant ratio had represented its major impact on flow behavior. It was observed that a lower BA concentration with high concentration of surfactant had a positive impact on flow behavior. The value of Carr's index ranged from 13 to 22 representing a better flow behavior of lipospheres. The results from angle of repose had also confirmed the excellent flow characteristics of lipospheres because most of the preparations exhibited angle of repose less than 25 [16, 18]. Similarly, outcomes of Hausner's ratio also confirmed the excellent flow behavior of lipospheres. It was less than 1.5 for all of the liposphere preparations indicating the excellent micromeritics [15, 21] which would be helpful in transformation of LS into a tablet or capsule dosage form.

For this study, a precise and sensitive HPLC method was efficiently established to quantify SG amount in controlled release LS selecting acetonitrile : methanol : phosphate buffer (40 : 40 : 20) as mobile phase (Figure 4).

The linearity curve was established at concentration range 06 to 30 ng/mL in mobile phase and at concentration range 10 to 50 ng/mL in plasma. In current study, a 10 *μ*L injection volume at flow rate of 1 mL/min was run for 10 minutes while maintaining a column pressure of 1400 psig at 212 nm detection wavelength. The retention time for SG were observed as 4.07 min in mobile phase and 4.18 min in plasma with a tailing factor of 1.56 was observed. The developed procedure was validated according to ICH guidelines where correlation coefficients (*R*^2^) for SG was found to be 0.9989 in mobile phase and 0.9998 in plasma in linearity analysis while during intraday and interday precision studies, %RSD was found to be <2%. The mean drug recovery in mobile phase and plasma was observed as 98.41% to 98.92% while the assay of optimized LS indicated 99.89% of SG contents. In mobile phase, the LOD and LOQ for SG were found to be 0.007 *μ*g/mL and 0.047 *μ*g/mL, respectively, while in plasma, the LOD and LOQ of SG were found to be 6.25 ng/mL and 7.02 ng/mL, respectively. Similarly, the %RSD for system suitability analysis was also observed to be <1% for SG. The established HPLC procedure method demonstrated itself accurate, cost-effective, and rapid for estimating SG contents both in liposphere formulation and plasma.

**Figure 5:**
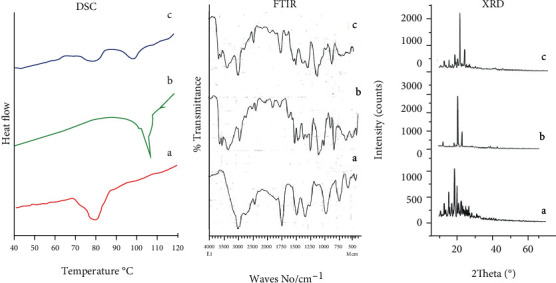
Thermograms of behenic acid (a), saxagliptin (b), and saxagliptin-loaded lipospheres (c); FTIR patterns of behenic acid (a), saxagliptin (b), and saxagliptin-loaded lipospheres (c); and XRD spectra of saxagliptin (c), behenic acid (b), and saxagliptin-loaded lipospheres (a).

FTIR study showed a good compatibility between SG and BA. The FTIR spectrum of formulated lipospheres was compared with the individual spectra of SG and BA. The FTIR spectra of SG, BA, and optimized LS were depicted in section FTIR of Figure 5. The characteristic Aliphatic C-N stretches were observed at 1519.77 cm^−1^ in the individual spectrum of SG and in the FTIR spectrum of SG-loaded lipospheres. The particular aromatic peaks of C-H functional groups of SG were also quite visible at 2919.18 cm^−1^ in the FTIR spectrum of SG-loaded lipospheres as shown in section FTIR-C of Figure 5. Shifting or absence of principal peaks of any functional groups of drug or BA was not observed neither in the spectrum of SG nor in the FTIR spectrum of lipospheres. This absence of any new peak in the FTIR spectrum of drug loaded prepared lipospheres depicting the intactness of saxagliptin in prepared lipospheres concluding that lipid polymer was found to be compatible for formulation of lipospheres of SG [26].

Differential scanning calorimetry was employed to check the compatibility of drug and BA. DSC thermograms of SG, BA, and SG-loaded lipospheres (OL) were depicted in Figure 5 section DSC. A sharp endothermic peak of drug was observed at a temperature of 107°C indicating melting of drug. The specific endothermic peak conforming the melting point of BA was clearly observed at a temperature of 80°C for BA alone (a) as depicted in Figure 5. In drug-loaded lipospheres, the specific peaks conforming to melting point of SG and BA were clearly visible at temperature of 107°C and 80°C, respectively, representing the compatibility of lipid with drug. The DSC findings suggested the lack of harmful impact of formulation conditions and process variables on drug stability [6, 25].

In current study, X-ray powder diffraction studies for SG, BA, and drug-loaded lipospheres were also conducted. Figure 5 section XRD indicated the XRD patterns of lipospheres along with those of BA and raw crystals of drug. Specific peaks of SG were detected at 2*θ* of 10°, 15°, 20°, and 28° representing the crystalline nature of SG as shown in Figure 5 XRD (c). The specific peaks of SG and BA were also clearly observed in Figure 5 for drug-loaded lipospheres. The analysis of XRD pattern of drug-loaded lipospheres (Figure 5) revealed sharp as well as scattered peaks depicting that during preparation of lipospheres, a fraction of SG was converted into amorphous form. The XRD patterns of lipospheres showed a mild reduction in peak intensities which suggested reduction in crystallinity of drug. In the diffraction position relevant to SG or BA, absence of any significant change was observed. The study revealed that preparation steps of drug-loaded lipospheres had not produced any negative effects on SG [25].

**Figure 6:**
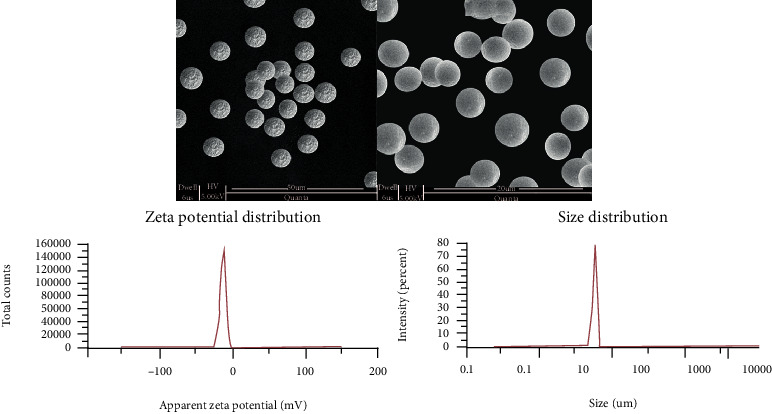
Microphotographs by SEM, zeta potential curve, and size distribution curve of optimized liposphere formulation.

Zeta potential, particle size, and size distribution of optimized liposphere formulation were determined with the help of Malvern Zetasizer. Size distribution and zeta potential curves of selected liposphere preparation were represented in Figure 6. Liposphere size distribution ranged from 11 to 20 *μ*m, while the major fraction (75%) of the lipospheres showed average size of 15 *μ*m. It was observed that particle size was greatly affected by polymer concentration (BA). Likewise, particle size of lipospheres was found to be greatly influenced by SS, and here, a higher speed of 3000 rpm was found to be optimum for mixing immiscible microparticles with aqueous phase and prevented aggregation of hydrophobic polymeric lipospheres [25]. T-80 concentration at a level of (1.50%) was also found to be a contributing factor towards the production of micron-size lipospheres because in the preliminary stage, liposphere preparation was failed without surfactant. Liposphere stability was deduced from zeta potential measurement which is defined as an electrical/charge potential at the shear plane. The lipospheres which had higher zeta potential were found to have good storage stability. Here, for LS formulation, zeta potential ranged from -2 mV to -32 mV as shown in Figure 6 section zeta potential distribution representing that lipospheres would have good storage stability. Negative charge presence in high intensity would produce electrostatic repulsion between particles leading to prevention of formation of aggregates of lipospheres [26]. Liposphere formulations were found to be spherical and discrete in their morphology as depicted in Figure 6.

The image of formulation of lipospheres was observed to check the impact of surfactant concentration, SS, and BA on lipospheres morphology. The SEM outcomes indicated that liposphere formulation were having uniform and smooth surface because of the fact that OL have higher T-80 concentrations with lower polymer (2.0%) concentration. In this study, surfactant was also found to have an important role not only in the production of smooth micron-sized lipospheres but also in emulsion droplet stabilization of BA [27]. Moreover, SEM image also revealed that higher stirring speed had also contributed to produce spherical lipospheres and prevented the formation of aggregates of hydrophobic lipid polymer [15, 27].

In stability studies, no significant changes were observed in drugs' contents, particle size, and PDI of OL. During stability analysis, the size of optimized LS was observed to be constant at a level of 20-50 *μ*m. Similarly, the PDI of OL was found to be as 0.422 and 0.423 at day 30 and day 90, while the drug contents at day 30 and day 90 were observed to be 99.08 and 98.98, respectively. The drug content and PDI were found to be not only satisfactory but also within official limits indicating the significant stability of optimized LS.

**Figure 7:**
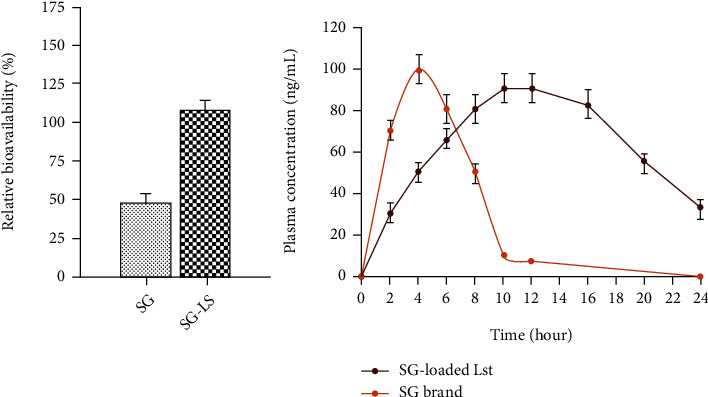
The relative bioavailability plots and plasma concentrations versus time curves for SG released from marketed brands and optimized lipospheres.

In pharmacokinetic evaluation, a significant decrease in maximum plasma concentration (*C*_max_) of SG (Figure 7) was observed from the optimized LS formulation.

The calculated *C*_max_ of SG from marketed brand and optimized LS were found to be 99.66 ± 2.97 ng/mL and 75.63 ± 3.85, respectively, as shown in Table 6. From the reference marketed brand, the SG was immediately released causing a lower time to achieve maximum plasma concentration (*T*_max_) which suggested that drug took less time to be absorbed. The SG released from marketed brand showed a *T*_max_ of 3.55 h. However, the optimized LS indicated a higher mean *T*_max_ value of 10.53 h for SG demonstrating comparatively a controlled release of SG. It is further suggested that drug took more time to attain *C*_max_ after releasing from LS as compared to SG released from selected reference brand. In literature, a similar increase in *T*_max_ and decrease in *C*_max_ for the modified release formulations of metformin (antidiabetic drug) and felodipine (antihypertensive drug) had also been observed as compared to immediate release formulation [11, 28]. The experimental variation in calculated *T*_max_ and *C*_max_ might be due to the difference of composition of optimized LS and reference brand.

The optimized LS showed a half-life (*t*_1/2_) of 17.32 ± 1.76 h for SG, while marketed brands indicated a *t*_1/2_ of 6.09 ± 1.39 h for SG. By using optimized lipospheres, the plasma drug concentration was effectively maintained for a prolonged time interval as apparent from a lower value of elimination rate constant (*K*_el_) 0.076 for SG-loaded LS as compared to saxagliptin marketed brand (0.359). It was quite clear that the elimination of SG from marketed brand took place within 4-5 h, while LS caused an increase in the SG elimination life up to 24 h which suggest the potential of LS regarding prolonged and controlled drug delivery. The administration of drug encapsulated in LS raised the MRT significantly. The observed MRT_0-24_ was found to be 9.5 h and 18.64 h for SG brand and SG-loaded LS which suggested a controlled drug release from LS. The fact could be attributed to the use of lipid polymer which caused a decreased in SG clearance from systemic circulation [11]. Hence, a controlled release function of OL could easily be concluded from the results of MRT that was found to be significantly (*p* < 0.05) higher for lipospheres as compared to marketed brand of SG. A pharmacokinetic parameter AUC (area under curve) is commonly employed to calculate bioavailability, and the enhancement in bioavailability is linked with an increase in AUC. In the current study, the AUC_0-24_ for SG reference brand and SG-loaded LS were calculated as 267.8 ± 9.52 and 10275.8 ± 6.53 ng.h.mL^−1^ respectively.

As compared to marketed brand, a higher value of AUC for LS was successfully achieved which was an indicative of enhanced absorption of SG in blood. The significant improvement in AUC suggested that SG released form LS very slowly for a prolonged time. The controlled release of SG could be associated with a very slow erosion of BA and difficult diffusion of drug through lipid macromolecule (BA). Furthermore, the administration of SG as encapsulated in LS brought a successful achievement of improved relative bioavailability for SG (Figure 7) as evident from higher AUC_0-24_ and *T*_max_. Consequently, a desired therapeutic response in diabetic patients at decreased dose with decrease dosing frequency could successfully be obtained by formulating SG-loaded LS which would enhance patients' compliance because of a reduction in drugs associated side effects along with maintaining the steady state plasma drug concentration over a prolonged time interval.

## 4. Conclusion

The negatively charged, micron-sized (10-50 *μ*m), smooth, and free flowing, spherical LS for the prolonged administration of SG was successfully formulated by simple convenient hot melt emulsion congealing process. By using Box-Bhenken design, different formulation parameters were efficiently optimized and statistically explicated. The LS had displayed maximum PY and EE with an effective control over *in vitro* release of SG for 12 h time interval. Saxagliptin had revealed appropriate compatibility with lipid macromolecule (BA) and exhibited its mild transformation from crystalline to amorphous form during encapsulation process. The applied behenic acid was emerged as a suitable carrier to accomplish the desired release pattern of SG but T-80 played a critical role because T-80 had greatly affected the SG released by regulating not only the production but also the morphology of the LS. The pharmacokinetic analysis had recommended a strong impact of LS formulations on *in vivo* behavior of SG. Furthermore, the encapsulation of SG into LS brings about a prolonged, slow *in vitro* release of SG with subsequent accomplishment of upgraded MRT, half-life, and AUC of SG. As compared to conventional oral tablet used as reference in current studies, the *in vivo* evaluation suggested a significant improvement in SG bioavailability with higher *T*_max_ and lower *C*_max_ because of slow and controlled release of medicament from optimized BA lipospheres. So, such a novel approach of formulation of LS from the safe biodegradable BA for administration of medicaments could bring noteworthy benefits for a chronic illness that demands frequent dosing of medicaments. At the same time, the current study requires the utilization of suitably a large size of population in order to analyze further the acceptance, competence, and suitability of LS in drug delivery. However, the current study has shown a potential to achieve improvement of patient's compliance by increasing the drug exposure in systemic circulation without increasing pill burden, dosing frequency, and drug dose.

## Figures and Tables

**Table 1:** Compositions of different liposphere formulations and the obtained outcomes of percentage yield, entrapment efficiency, and SG release from lipospheres.

Formulations	Formulation components (actual level % *w*/*v*)			Responses (average value% ± SD)		
	BA	T-80	SS	PY	EE	DR
F1	2.00	1.50	3000	70 ± 3.18	66 ± 2.39	51 ± 2.31
F2	3.00	0.50	2250	41 ± 1.93	49 ± 2.67	77 ± 2.63
F3	2.00	0.50	1500	32 ± 2.05	40 ± 3.26	83 ± 2.35
F4	3.00	1.50	2250	59 ± 3.89	55 ± 1.87	56 ± 1.96
F5	2.00	1.00	2250	60 ± 1.97	59 ± 1.91	71 ± 3.77
F6	1.00	1.00	1500	67 ± 3.36	47 ± 3.73	82 ± 2.58
F7	1.00	0.50	2250	63 ± 2.58	41 ± 2.57	67 ± 1.88
F8	2.00	0.50	3000	37 ± 3.73	52 ± 3.14	79 ± 2.39
F9	2.00	1.00	2250	60 ± 1.78	59 ± 2.65	72 ± 3.76
F10	2.00	1.50	1500	51 ± 3.21	46 ± 3.16	70 ± 2.39
F11	2.00	1.00	2250	60 ± 2.64	59 ± 1.34	72 ± 1.84
F12	1.00	1.50	2250	65 ± 2.19	70 ± 2.67	42 ± 2.19
F13	2.00	1.00	2250	60 ± 2.18	59 ± 3.07	72 ± 3.39
F14	3.00	1.00	3000	47 ± 3.53	38 ± 3.09	72 ± 2.25
F15	3.00	1.00	1500	34 ± 3.76	30 ± 2.31	90 ± 3.13
F16	2.00	1.00	2250	60 ± 1.87	59 ± 1.59	72 ± 2.71
F17	1.00	1.00	3000	66 ± 3.25	62 ± 1.98	59 ± 2.44

*α*: 1.681; BA: behenic acid; SS: stirring speed; PY: percentage yield; EE: entrapment efficiency; DR: drug release, *n* = 3.

**Table 2:** The regression analysis of percentage yield, entrapment efficiency, and SG release.

Parameters	PY	EE	DR
*R*-squared	0.9078	0.9031	0.9805
Adj *R*-squared	0.7893	0.8425	0.9554
Pred *R*-squared	0.8568	0.7653	0.7929
Adeq precision	21.638	25.314	23.067
Std. dev.	5.58	4.11	2.55
Mean	54.82	52.41	69.82
C.V. %	10.18	9.75	3.65
PRESS	348.21	298.52	717.25

**Table 3:** Results of analysis of variance (ANOVA) for PY, EE, and DR with their corresponding *p* value.

Source	Percentage yield (*Y*_1_)		EE (*Y*_2_)		DR (*Y*_3_)	
	*F* value	*p* value prob > *F*	*F* value	*p* value prob > *F*	*F* value	*p* value prob > *F*
Model	11.43	0.0032	9.36	0.0038	39.08	<0.0001
BA	25.69	0.0014	11.09	0.0033	38.08	0.0004
T-80	22.82	0.0019	14.48	0.0029	145.04	<0.0001
SS	15.96	0.0026	14.48	0.0029	81.28	<0.0001
*X*_1_ *X*_2_	9.03	0.0481	5.07	0.0591	8.61	0.0047
*X*_1_ *X*_3_	4.46	0.0580	2.07	0.2712	9.27	0.037
*X*_2_ *X*_3_	3.58	0.0253	3.47	0.2982	13.13	0.0017
*X*_1_^2^	5.31	0.0614	8.08	0.0041	5.09	0.0593
*X*_2_^2^	13.07	0.0029	2.39	0.0044	43.65	0.0002
*X*_3_^2^	16.38	0.0021	12.35	0.0092	32.62	0.0006
LOF	217.26	0.0008	182.53	0.0004	55.58	0.0193

**Table 4:** Composition, predicted versus observed results, size, zeta potential, desirability factor, and prediction errors (PE) of optimized lipospheres formulation.

OL composition		Response	Observed value	Predicted value	PE	Desirability	Size (*μ*m)	ZP (mv)
BA (%)	2.5	PY	80	82	3.49	0.993	20 ± 1.32	−32 ± 2.16
T-80 (%)	1.5	EE	78	80	4.91	0.936		
SS (rpm)	3500	SG-Rel	40	38.5	4.52	0.745		

SS: stirring speed; Rel: release; EE: entrapment efficiency; OL: optimized lipospheres; PE: prediction error; ZP: zeta potential.

**Table 5:** The outcomes of flow properties, LS size, zeta potential, and polydispersity index of SG-loaded lipospheres.

Formulations	Micromeritics			Size (*μ*m)	ZP (mv)	PDI
	Angle of repose	Hausner's ratio	Carr's index			
F1	18 ± 2.63	1.04 ± 2.93	14 ± 2.19	21 ± 2.26	31 ± 4.26	0.543 ± 1.09
F2	23 ± 2.08	1.18 ± 2.64	20 ± 1.64	40 ± 2.76	39 ± 3.98	0.452 ± 0.97
F3	24 ± 2.07	1.21 ± 1.55	22 ± 2.13	50 ± 3.83	37 ± 4.86	0.542 ± 1.07
F4	17 ± 2.87	1.03 ± 2.37	13 ± 3.25	38 ± 3.37	35 ± 5.49	0.491 ± 0.98
F5	20 ± 1.59	1.09 ± 2.92	16 ± 1.68	35 ± 2.59	33 ± 3.83	0.475 ± 1.03
F6	18 ± 2.91	1.03 ± 1.63	13 ± 2.66	29 ± 3.41	31 ± 4.78	0.429 ± 1.02
F7	19 ± 1.87	1.06 ± 1.87	15 ± 2.62	31 ± 4.38	33 ± 5.28	0.576 ± 0.87
F8	23 ± 2.62	1.16 ± 2.28	19 ± 1.47	47 ± 3.65	40 ± 3.75	0.511 ± 0.88
F9	20 ± 2.33	1.08 ± 2.95	16 ± 1.92	35 ± 3.76	32 ± 4.81	0.522 ± 1.04
F10	22 ± 3.18	1.13 ± 3.15	17 ± 2.15	38 ± 2.89	36 ± 3.43	0.475 ± 0.97
F11	20 ± 2.71	1.09 ± 2.07	15 ± 1.67	35 ± 3.64	33 ± 3.62	0.525 ± 1.16
F12	17 ± 1.94	1.02 ± 2.65	13 ± 1.78	28 ± 4.87	30 ± 3.37	0.495 ± 0.94
F13	20 ± 1.83	1.08 ± 3.09	14 ± 1.51	35 ± 2.82	32 ± 5.88	0.532 ± 1.09
F14	24 ± 2.95	1.19 ± 2.29	20 ± 2.13	41 ± 2.21	37 ± 4.52	0.448 ± 0.89
F15	25 ± 2.66	1.22 ± 2.32	22 ± 3.08	48 ± 3.42	41 ± 3.84	0.489 ± 1.04
F16	19 ± 2.81	1.09 ± 2.79	16 ± 1.87	35 ± 3.79	32 ± 4.42	0.434 ± 0.97
F17	18 ± 1.29	1.06 ± 3.03	15 ± 2.39	25 ± 4.68	29 ± 4.92	0.517 ± 1.12

PDI: polydispersity index; ZP: zeta potential.

**Table 6:** The results of studied pharmacokinetic parameters of SG-loaded optimized lipospheres and SG reference brand.

Pharmacokinetic variables	Group A SG-OL	Group B SG-brand
*T*_max_ (h)	10.53 ± 3.56	3.55 ± 2.18
*C*_max_ (ng/mL)	75.63 ± 3.85	99.66 ± 2.97
MRT_0-24_ (h)	18.64 ± 3.18	9.5 ± 4.54
AUC_0-24_ (ng.h/mL)	10275.8 ± 6.53	267.8 ± 9.52
*t*_1/2_ (h)	17.32 ± 1.76	6.08 ± 1.39
*K*_el_ (h-1)	0.076 ± 1.072	0.359 ± 0.88
AUC_0-∞_ (ng.h/mL)	14579.7 ± 42.68	738.4 ± 13.76
AUMC_0-∞_ (ng.h/mL)	238485.6 ± 13.34	55247.7 ± 12.18

## Data Availability

Data could be provided from coauthor Dr. Safirah Maheen (safirah.maheen@uos.edu.pk) upon request.
